# Genotoxicity of methyl acrylate and ethyl acrylate and its relationship with glutathione

**DOI:** 10.1007/s00204-022-03322-1

**Published:** 2022-06-15

**Authors:** F. Oesch, N. Honarvar, E. Fabian, L. Finch, S. Hindle, K. Wiench, R. Landsiedel

**Affiliations:** 1 Oesch-Tox Toxicological Consulting and Expert Opinions, 55263 Ingelheim, Germany; 2 Institute of Toxicology, Johannes Gutenberg-University of Mainz, Mainz, Germany; 3 BASF SE, Experimental Toxicology and Ecology, 67056 Ludwigshafen am Rhein, Germany; 4 Arkema Inc., Philadelphia, PA USA; 5 The DOW Chemical Company, Horgen, Switzerland; 6 Regulatory Toxicology of Chemicals, BASF SE, 67056 Ludwigshafen am Rhein, Germany; 7 Pharmacy, Pharmacology and Toxicology, Free University of Berlin, Berlin, Germany

**Keywords:** Methyl acrylate, Ethyl acrylate, Mutagenicity, Glutathione

## Abstract

Methyl acrylate (MA) and ethyl acrylate (EA) had previously tested positive for mutagenicity in vitro, but in vivo studies were negative. One of the metabolism pathways of alkyl acrylates is conjugation with glutathione. The glutathione availability is restricted in standard in vitro test systems so that they do not reflect the in vivo metabolism in this respect. We investigated whether the addition of glutathione to the in vitro L5178Y/TK^+/−^ mouse lymphoma mutagenicity test prevents alkyl acrylate’s mutagenicity in vitro. We also investigated whether the quantitative relationships support the notion that the GSH supplemented in vitro systems reflect the true in vivo activity. Indeed, glutathione concentrations as low as 1 mM completely negate the mutagenicity of MA and EA in the L5178Y/TK^+/−^ mouse lymphoma mutagenicity test up to the highest concentrations of the two acrylates tested, 35 µg/ml, a higher concentration than that previously found to be mutagenic in this test (14 µg MA/ml and 20 µg EA/ml). 1 mM Glutathione reduced the residual MA and EA at the end of the exposure period in the mutagenicity tests by 96–97%, but in vivo up to 100 mg/kg body weight MA and EA left the glutathione levels in the mouse liver and forestomach completely intact. It is concluded that the in-situ levels of glutathione, 7.55 ± 0.57 and 2.84 ± 0.22 µmol/g mouse liver and forestomach, respectively, can efficiently protect against MA and EA-induced mutagenicity up to the high concentration of 100 mg MA and EA/kg body weight and that the negative in vivo mutagenicity tests on MA and EA reflect the true in vivo situation.

## Introduction

Methyl acrylate (MA) and ethyl acrylate (EA) are important monomers used for the production of polymers and copolymers which in turn are used for the production of numerous end products including coatings, varnishes, adhesives, food packaging and cosmetics. Interestingly, MA and EA naturally occur in pineapples and EA also in further fruits such as raspberries and blackberries and in cheese (Suh et al. 2018).

Previous studies on the potential genotoxicity of MA and EA showed activities in vitro, but not in vivo.

MA and EA were negative in bacterial mutation tests (Florin et al. 1980; Suh et al. 2018), but at cytotoxic concentrations MA and EA were positive in mammalian cell tests, the in vitro micronucleus test in CHO cells (Kirpnick et al. 2005) and the mouse lymphoma TK^+/−^ test (Suh et al. 2018). In the in vivo micronucleus test in mice MA was negative upon inhalation or oral application (Hachiya et al. 1982, Sofuni et al. 1984). In the in vivo transgenic gpt and Spi^−^ oral mutagenicity test in mice EA also was negative. However, MA and EA were reported as positive in the in vivo micronucleus test in mice upon intraperitoneal injection (Przybojewska et al. 1984). OECD TG 474 does not recommend this route of application for the micronucleus test (OECD 2012). Further shortcomings of this test by Przybojewska et al. (1984) include a lack of dose–response despite a large dose range (37.5–300 mg/kg) and a lack of identification of the purity of the tested substance. Moore et al. (1988) had shown that in alkyl acrylate samples methyl hydroquinone was present as an inhibitor of spontaneous reactions. This substance has been tested by NTP in the mouse lymphoma assay and positive results were obtained at concentrations as low as 1.25 µg/ml). Furthermore, in an attempt by Ashby et al. (1989) to repeat the positive in vivo micronucleus test on EA by Przybojewska et al. (1984), it was not reproducible.

All things considered, the positive in vitro mutagenicity tests at cytotoxic concentrations of MA and EA were not confirmed in vivo.

Metabolism studies had shown that the two major metabolism pathways for MA and EA are hydrolysis and conjugation with glutathione (GSH). An obvious possibility for the negative in vivo genotoxicity tests for MA and EA, therefore, is an efficient metabolic elimination by GSH under physiological conditions. The present study was undertaken to assess whether the addition of GSH to the in vitro genotoxicity tests on MA and EA is able to abrogate the in vitro genotoxicity and, hence correctly predicts the lack of genotoxicity in vivo.

## Materials and methods

### Chemicals

Methyl acrylate, CAS No. 96-33-3, batch 012017eda0, 99.93% pure. The stability of the test substance under storage conditions (refrigerator, light exclusion) over the test period was guaranteed by the manufacturer—Ethyl acrylate, CAS No. 140–88-5, batch 013948eda0, 99.92% purity. The stability of the test substance under storage conditions (refrigerator, light exclusion) over the test period was guaranteed by the manufacturer. Glutathione (GSH) was obtained from Sigma, methyl methane sulfonate (MMS) was obtained from Merck-Schuchardt.

### Cell culture

#### Cell line

The mouse lymphoma L5178 TK^+/−^ (clone 3.7.2c) (Mitchell et al. 1997) was used. This cell line is distinguished by the combination of a high proliferation rate (doubling time of about 9–10 h), high plating efficiency (about 90%) and a stable karyotype with a near diploid number of 40 ± 1 chromosomes. Stocks of the cell line were stored in 1-ml portions at −196 °C in the gas phase above the liquified nitrogen using 7% (v/v) dimethylsulfoxide (DMSO) in fetal calf serum (FCS) as a cryoprotectant. Each batch used for mutagenicity testing was checked for mycoplasma contamination.

#### Culture media

The basic culture medium (“RPMI-0”) was RPMI 1640 including glutamine supplemented with 1% (v/v) penicillin (10,000 IU), 1% streptomycin (10,000 µg/ml) and 1% (v/v) sodium pyruvate (10 mM). For washing of cells, RPMI-0 was supplemented with 5% (v/v) FCS (“RPMI-5”). For treatment with the test substance and for subculturing RPMI-0 was supplemented with 10% (v/v) FCS (“RPMI-10”), for determination of the cloning efficiency and selection it was supplemented with 20% (v/v) FCS (“RPMI-20”).

Pretreatment medium A ("THMG" medium): RPMI-10 supplemented with 3 µg/ml thymidine, 5 µg/ml hypoxanthine, 0.1 µg/ml methotrexate and 7.5 µg/ml glycine.

Pretreatment medium B ("THG" medium): RPMI-10 supplemented with 3 µg/ml thymidine, 5 µg/ml hypoxanthine and 7.5 µg/ml glycine.

Selection medium ("TFT" medium) RPMI-20 supplemented with 4 µg/ml trifluoro-thymidine.

#### Culture

Deep-frozen stock cultures were thawed at 37 °C in a water bath, and volumes of 1 ml were transferred into 25 cm2 flasks containing 10 ml of the above-described RPMI-10 medium. After incubation for about one day, the cells were centrifuged at 800–1000 rpm (134–173 g) for 5 min. Subsequently, the medium was removed and the cells were resuspended in 20 ml RPMI-10 medium, transferred to 75 cm^2^ flasks, sub-cultured twice weekly and kept under 5% (v/v) C0_2_ at 37 °C and ≥ 90% relative humidity.

### Cytotoxicity

#### Test procedure

3 × 10^5^ exponentially growing cells in 30 ml per treatment group were incubated in 75 cm^2^ flasks 4–5 days prior to the start of the experiment. Following centrifugation and resuspension 1.5 × 10^7^ cells per culture were dispensed into 75 cm^2^ flasks. Two cultures were treated in parallel for each test group. The treatment medium was added and the cultures were incubated for a 4-h exposure period. As shown in Tables 1 and 2 the cells were exposed to several concentrations of the test substance (1) alone or (2) in the presence of 10 mM GSH or (3) followed by 10 mM GSH or (4) in presence of 10 mM 2-mercaptoethanol.

**Table 1:** Cytotoxicity of MA to MLA cells abrogated by 10 mM GSH

Methyl acrylate (µg/ml)	Methyl-acrylate only			Methyl acrylate + 10 mM glutathione simultaneously			Methyl acrylate treatment followed by posttreatment with 10 mM glutathione			Methyl acrylate + 10 µM 2-mercaptoethanol		
	RGDT	RSG	RTG	RGDT	RSG	RTG	RGDT	RSG	RTG	RGDT	RSG	RTG
0 (Vehicle control)	100.0	100.0	**100.0**	100.0	100.0	**100.0**	100.0	100.0	**100.0**	100.0	100.0	**100.0**
1.0	107.0	90.3	**101.6**	99.7	85.7	**67.3**	93.2	84.1	**90.8**	101.3	101.3	**94.6**
5.0	103.1	68.3	**74.2**	99.6	85.5	**75.3**	85.8	62.7	**68.8**	102.0	82.1	**82.1**
10.0	99.7	61.0	**59.0**	98.8	90.8	**82.8**	86.4	59.5	**73.2**	98.0	81.6	**73.8**
15.0	94.2	49.5	**50.3**	104.8	87.0	**92.4**	84.2	59.1	**60.0**	94.7	70.7	**80.4**
20.0	89.5	46.5	**38.1**	96.3	79.9	**68.1**	79.1	42.0	**55.4**	84.2	56.0	**49.0**
25.0	874	25.7	**27.0**	108.1	83.5	**74.8**	73.3	10.1	**6.2**	86.4	41.3	**52.1**
30.0	88.5	n.c	n.c	100.5	89.6	**91.4**	833	15.6	**13.5**	90.3	37.0	**28.2**
35.0	88.2	n.c	n.c	103.4	77 1	**66.8**	841	11.0	**6.2**	86.3	48.0	**46.4**

*RGDT* relative growth during treatment [%]; *RSG* relative suspension growth [%]; *RTG* relative total growth [%]; *n.c.* culture not continued due to strong cytotoxicity

**Table 2:** Cytotoxicity of EA to MLA cells abrogated by 10 mM GSH

Ethyl acrylate (µg/ml)	Ethyl acrylate only			Ethyl acrylate + 10 mM glutathione simultaneously			Ethyl acrylate treatment followed by posttreatment with 10 mM glutathione			Ethyl acrylate + 10 µM 2-mercaptoethanol		
	RGDT	RSG	RTG	RGDT	RSG	RTG	RGDT	RSG	RTG	RGDT	RSG	RTG
0 (Vehicle control)	100.0	100.0	**100.0**	100.0	100.0	**100.0**	100.0	100.0	**100.0**	100.0	100.0	**100.0**
1.0	101.0	102.2	**128.7**	88.6	87.3	**75.6**	99.9	97.9	**78.6**	89.5	106.9	**97.5**
5.0	82.1	79.6	**112**	77.5	79.5	**68.8**	88.3	86.0	**78.0**	89.3	64.9	**69.2**
10.0	74.2	65.5	**68.6**	87.6	81.9	**68.6**	86.1	76.1	**71.3**	86.5	87.3	**87.3**
15.0	70.9	57.1i	**58.9**	92.8	75.8	**65.6**	81.2	65.6	**55.9**	83.9	71.9	**73.1**
20.0		45.7				**78.0**	77.5	62.5				
25.0	67.5	37.3	**37.9**	87.3	83.1	**62.2**	**74.7**	43.8	**37.3**	86.4	72.9	**70.7**
30.0	61.5	17.5	**14.2**	89.5	83.2	**69.7**	82.3	17.2	**7.5**	79.3	66.5	**51.7**
35.0	59.3	6.6	!**1.9**	95.7	86.4	**76.1**	80.8	nc	nc	76.4	41.8	**42.4**

*RGDT* relative growth during treatment [%]; *RSG* relative suspension growth [%]; *RTG* relative total growth [%]; *n.c.* culture not continued due to strong cytotoxicity

At the end of the exposure period, the cells were transferred to tubes, centrifuged for 5 min at 1000 rpm (173 g) and resuspended in RPMl-5 medium. The washing of the cells was repeated at least once. Then the cells were again centrifuged at 1000 rpm (173 g, 5 min) and resuspended in RPMl-10 medium. From each culture, a sample of treated cells (2 × 10^5^ cells/ml or 6 × 10^6^ cells/flask) was pipetted in 75 cm^2^ flasks and incubated for a 2-day expression period. To maintain exponential growth during this phase, each culture was counted daily and the cell numbers were adjusted every day to 2 × 10^5^ cells/ml in 30 ml RPMl-10 medium. The cell numbers were determined using a cell counter (CASY®, Roche Applied Science, Mannheim, Germany).

#### Cytotoxicity determinations

##### Cloning efficiency 1 (survival)

At the end of the exposure period, the cells were centrifuged (134–173 g, 5 min) and 400 cells from each test group were resuspended in 50 ml RPMl-20 medium (8 cells/ml). Per culture 200 µl were dispensed in each well of two 96-well plates (1.6 cells/well). After incubation for 9–11 days the plates were scored for empty wells.

##### Cloning efficiency 2 (viability)

After the expression period, 2 days after the end of exposure, the cells were centrifuged (134–173 g, 5 min) and 400 cells from each culture were resuspended in 50 ml RPMl-20 medium (8 cells/ml). Per culture 200 µl were dispensed in each well of two 96-well plates (1.6 cells/well). After incubation, for at least 9 days the plates were scored for empty wells.

##### Relative suspension growth and relative total growth

For calculation of the relative suspension growth (RSG) and the relative total growth (RTG) the cell counts determined within the expression period at second and third passage after exposure in the case of 4-h exposure and first, second and third passage after exposure in the case of 24-h exposure were used.

The cloning efficiency (CE, %) and the relative cloning efficiency (RCE, %) were calculated as follows:

$${\text{CE}} = \frac{{ - \ln \frac{{{\text{total}}\;{\text{number}}\;{\text{of}}\;{\text{empty}}\;{\text{wells}}}}{{{\text{total}}\;{\text{number}}\;{\text{of}}\;{\text{seeded}}\;{\text{wells}}\;(96)}}}}{{{\text{number}}\;{\text{of}}\;{\text{seeded}}\;{\text{cells}}\;{\text{per}}\;{\text{well}}\;(1.6)}} \times 100$$

$${\text{RCE}} = \frac{{{\text{CE}}\;{\text{of}}\;{\text{the}}\;{\text{test}}\;{\text{group}}}}{{{\text{CE}}\;{\text{of}}\;{\text{the}}\;{\text{vehicle}}\;{\text{control}}}} \times 100$$

The relative growth during treatment (RGDT, %), the total suspension growth after 4 h and after 24 h exposure (SG), the relative suspension growth (RSG, %) and the relative total growth (RTG, %) were calculated as follows:

$${\text{RGDT}} = \frac{{{\text{Cell}}\;{\text{count}}\;{\text{of}}\;{\text{the}}\;{\text{test}}\;{\text{group}}\;{\text{after}}\;{4}\;{\text{h}}\;{\text{treatment}}}}{{{\text{Cell}}\;{\text{count}}\;{\text{of}}\;{\text{the}}\;{\text{vehicle}}\;{\text{control}}\;{\text{after}}\;4\;{\text{h}}\;{\text{treatment}}}} \times 100$$

Total suspension growth after 4 h exposure:

$${\text{SG }} = \frac{{{\text{Cell}}\;{\text{count}}\;{\text{after}}\;24\;{\text{h}}}}{{2 \times 10^{5} \;{\text{cells}}\;{\text{per}}\;{\text{ml}}^{[1,2]} }} \times \frac{{{\text{Cell}}\;{\text{count}}\;{\text{after}}\;48\;{\text{h}}}}{{2 \times 10^{5} \;{\text{cells}}\;{\text{per}}\;{\text{ml}}^{[2]} }} \times \frac{{{\text{RGDT}}}}{100}$$

Total suspension growth after 24 h exposure:

$${\text{SG}} = \frac{{{\text{Cell}}\;{\text{count}}\;{\text{after}}\;24\;{\text{h}}}}{{2 \times 10^{5} \;{\text{cells}}\;{\text{per}}\;{\text{ml}}}} \times \frac{{{\text{Cell}}\;{\text{count}}\;{\text{after}}\;48\;{\text{h}}}}{{2 \times 10^{5} \;{\text{cells}}\;{\text{per}}\;{\text{ml}}^{[2]} }} \times \frac{{{\text{Cell}}\;{\text{count}}\;{\text{after}}\;{72}\;{\text{h}}}}{{{2} \times {10}^{{5}} \;{\text{cells}}\;{\text{per}}\;{\text{ml}}^{{[2]}} }}$$

$${\text{RSG}} = \frac{{{\text{SG}}\;{\text{of}}\;{\text{the}}\;{\text{test}}\;{\text{group}}}}{{{\text{SG}}\;{\text{of}}\;{\text{the}}\;{\text{vehicle}}\;{\text{control}}}} \times 100$$

$${\text{RTG}} = \frac{{{\text{RSG }} \times {\text{RCE}}_{2} }}{100}$$

where [1] cell number seeded following 4-h treatment and [2] if cell number was lower than 2 × 10^5^ cells per ml all remaining cells were seeded.

### Mutagenicity

#### Mutagenicity test procedure

3 × 10^5^ exponentially growing cells in 30 ml per treatment group were incubated in 75 cm^2^ flasks 4–5 days prior to the start of the experiment. During the week prior to treatment, spontaneous TK-deficient mutants (TK−/−) were eliminated from the stock cultures by incubating 3 × 10^5^ cells per 75 cm^2^ flask in a total volume of 30 ml for 1 day in “THMG" medium (pretreatment medium A), and for the following 3 days in “THG" medium (pretreatment medium B).

*Treatment and expression period.* Following centrifugation and resuspension 1.5 × 10^7^ cells per culture were dispensed into 75 cm^2^ flasks. Subsequently, the treatment medium was added, 19.6 ml RPMI-10 medium and 0.2 ml vehicle (DMSO, final concentration in the culture medium 1% v/v) alone or vehicle containing the test acrylate or vehicle containing the positive control substances 15 µg/ml MMS) and finally 0.2 ml ultrapure water containing GSH (final concentration 1 mM). Two cultures were treated for 4 h in parallel for each test group. At the end of the exposure period, the cells were transferred into tubes, centrifuged for 5 min at 800 rpm (134 g) and was resuspended in RPMI-5 medium. The washing of the cells was repeated at least once. Then the cells were centrifuged at 800 rpm (134 g, 5 min) and resuspended in RPMI-10 medium. From each culture, a sample of treated cells (2 × 10^5^ cells/ml or 6 × 10^6^ cells/flask) was pipetted into 75 cm^2^ flasks and incubated for a 2-day expression period. To maintain exponential growth during this phase, each culture was counted daily and the cell numbers were adjusted each day to 2 × 10^5^ cells/ml in 30 ml RPMI-10 medium. The cell numbers were determined using a cell counter (CASY, Roche Applied Science, Mannheim, Germany).

*Selection period*. For selecting the mutants the cells were centrifuged (134 g, 5 min) and 5 × 10^5^ cells from each culture were resuspended in 50 ml selection medium (“TFT" medium; 1 × 10^4^ cells/ml). Per culture 200 µl were dispensed in each well of two 96-well plates (2000 cells/well). After incubation for at least 9 days, the number of negative wells and the number of wells containing small or large colonies were scored for calculation of the mutant frequency (MF).

*Treatment conditions.* The pH and the osmolality were measured at least for the top concentration (with the addition of 1 mM glutathione) and for the vehicle controls (without the addition of 1 mM glutathione) at the beginning of treatment. Test substance precipitation was checked with the unaided eye at the end of the treatment period.

#### Determinations of mutant frequency

The number of empty wells and the number of wells containing colonies were scored. The colonies are classified into large colonies (fast growth: indication of a gene mutation) and small colonies (slow growth: indication of chromosome breakage). Small colonies are defined as less than 1/4 of the diameter of the well. Size is used as the key classification determinant. Morphology (the optical density of the small colonies is considerably higher) is used as a supporting determinant.

*The uncorrected mutant frequency* per 10^6^ cells (MFuncorr.) was calculated as follows:

$${\text{MF}}\;{\text{uncorr}}{.} = \frac{{ - \ln \frac{{{\text{total}}\;{\text{number}}\;{\text{of}}\;{\text{empty}}\;{\text{wells}}}}{{{\text{total}}\;{\text{number}}\;{\text{of}}\;{\text{seeded}}\;{\text{wells}}\;({96})}}}}{{{\text{number}}\;{\text{of}}\;{\text{seeded}}\;{\text{cells}}\;{\text{per}}\;{\text{well}}\;(2000)}} \times 10^{6}$$

*The corrected mutation frequency* (MFcorr.) was calculated taking the values of CE_2_ (see above under determinations of cytotoxicity) into account:

$${\text{MF}}_{{{\text{corr}}{.}}} = \frac{{{\text{MF}}_{{{\text{uncorr}}{.}}} }}{{{\text{CE}}_{2} }} \times 100$$

*Determination of borderline mutant frequency based on the global evaluation factor GEF.* This method requires that the MF exceeds a value based on the global distribution of the background MF of the test method (Moore et al. 2003, 2006, 2007). The GEF is defined as the mean of the negative/vehicle MF distribution plus one standard deviation. Based on a large database (*n* = 493 experiments) from six laboratories a GEF of 126 mutant colonies per 10^6^ cells (mean MFcorr = 99 × 10^–6^ colonies; standard deviation = 27 × 10^–6^ colonies) was calculated for the microwell method (Moore et al. 2006). To be judged positive the MF has to exceed a threshold of 126 colonies per 10^6^ cells above the concurrent negative/vehicle control value. Thus, the borderline mutant frequency was calculated for each experiment separately as follows:

$${\text{Borderline}}\;{\text{MF }} = {\text{MF}}\;_{{{\text{vehicle}}\;{\text{control}}\;{\text{corr}}}} + {\text{GEF}}\;{(}126 \times 10^{ - 6} {)}$$

#### Acceptance criteria

The ML TK^+/−^ assay is considered valid if the following criteria are met considering the international guidelines and the current recommendations of the IWGT (Moore et al. 2000, 2002, 2003, 2006, 2007):

- The absolute cloning efficiency obtained at the time of mutant selection (CE2) of the negative/vehicle controls should fall in the range of 65–120%.
- The suspension growth (SG) of the negative/vehicle controls referring to the expression period following treatment should fall in the range of 8–32 for 4-h exposure and 32–180 for 24-h exposure.
- The mutant frequency of the negative/vehicle controls should fall within the range of 50–170 × 10^–6^ colonies.
- The positive controls should yield an absolute increase in total MF that is an increase above the spontaneous background MF (an induced MF) of at least 300 × 10^–6^ colonies. The small colony MF should account for at least 40% of that IMF, which means a small colony IMF of at least 120 × 10^–6^ colonies. Alternatively, the positive controls should induce at least 150 × 10^–6^ small colonies above the spontaneous background MF. The upper limit of cytotoxicity observed in the positive controls should have a RTG that is greater than 10%.
- The highest applied concentration of the test substance should be 2 mg/ml, 2 µl/ml or 10 mM unless limited by cytotoxicity or solubility of the test substance. If toxicity occurs, the highest concentration should lower the RTG to 10–20% of survival. If precipitation occurs, the highest evaluated concentration should be the lowest concentration where precipitation is observed by the unaided eye.

#### Assessment criteria

The test substance is considered mutagenic if all the following criteria are met (Moore et al. 2006, 2007):

- The mutation frequency exceeds a threshold of 126 mutant colonies per 10^6^ cells (see above: GEF: Global Evaluation Factor) above the concurrent negative/vehicle control value and
- evidence of reproducibility of any increase in mutant frequencies, i.e. the mutagenic response occurs at least in both parallel cultures of one experiment and
- a statistically significant dose-related increase in mutant frequencies using an appropriate statistical trend test.

The test substance is considered non-mutagenic if at least one of the following criteria is met (Moore et al. 2006; 2007):

- The mutation frequency is below a threshold of 126 mutant colonies per 10^6^ cells (GEF) above the concurrent negative/vehicle control value or
- no evidence of reproducibility of an increase in mutant frequencies is obtained or
- no statistically significant dose-related increase in mutant frequencies using an appropriate statistical trend test is observed.

In the evaluation of the test results, the historical variability of the mutation rates in negative and vehicle controls (95% control limit) and the mutation rates of all negative and vehicle controls of this study was taken into consideration.

Results of test groups were rejected if the RTG was less than 10% of the respective negative/vehicle control.

Whenever a test substance is considered mutagenic according to the above-mentioned criteria, the ratio of small versus large colonies is used to differentiate point mutations from clastogenic effects. If the increase of the mutation frequency is accompanied by a reproducible and dose-related shift in the ratio of small versus large colonies clastogenic effects are indicated.

#### Statistics

An appropriate statistical method to test for linear trend (MS EXCEL function RGP) was performed to assess a possible linear dose-relation in mutant frequencies. The dependent variable was the corrected mutant frequency and the independent variable was the concentration. A trend was judged as statistically significant whenever the one-sided *p*-value was below 0.05 and the slope was greater than 0.

However, both, biological and statistical significance have been considered together.

### In vivo experiments

#### Animals

Male C57BL/6 mice, 7–9 weeks old, from Charles River Laboratories, Research Models and Services, Sulzfeld, Germany were acclimatized at least 5 days before the beginning of the dosing. The health status of the animals was checked at least once daily. No mortality or morbidity occurred during the study period. The animals were held at 20–24 °C and 45–65% relative humidity under a cycle of 12 h light (6:00–18:00 h) followed by 12 h darkness (18:00–6:00 h) individually in a Polycarbonate Cage (type III) on dust-free wooden bedding. They were fed with Standardized pelleted feed from Granovit AG, Kaiseraugst, Switzerland and had access to tap water ad libidum.

#### Administration of test material to the animals

The test material was administered to the mice in 10 ml water/kg body weight once orally by gavage.

#### Preparation of tissues

Three hours after administration of the test materials the mice were killed and the forestomach and the liver were removed from the animals. The large liver lobe was resected from the rest of the liver and a section of the large liver lobe was used for the experiments described in this publication.

#### Analytical determinations

Glutathione was determined photometrically using the method of Ellman (1959). MA and EA were analyzed by headspace-GC–MS according to the procedure described in ISO 20595, a method for volatiles in water. An aliquot of the culture supernatant/the cell pellet suspension was diluted to a volume of 5 ml in water in a 10-ml-headspace vial. 3 g of sodium sulfate and an internal standard solution were added to the vial. The capped vial was heated to 90 °C under constant agitation for 10 min. Subsequently, 1 ml of the headspace gas phase was injected into a GC–MS system (split-injection). MA and EA were quantitated in the SIM mode by the internal standard method.

## Results and discussion

### Cytotoxicity of MA and EA to mouse lymphoma (ML) cells abrogated by GSH

[MA/ml is cytotoxic to mouse lymphoma (ML) cells, which is abrogated by GSH]

Table 1 shows that MA is cytotoxic to ML L5178 cells. 10–25 µg MA/ml led to a relative total growth (RTG) between 27% to 59%, while 30–35 µg MA/ml led to a degree of cytotoxicity precluding continuation of the culture.

Simultaneous presence of 10 mM GSH in the medium practically abrogated this cytotoxicity and was especially visible at the concentration of 30 µg MA/ml, where the nominal relative total growth was back to 91% versus cytotoxicity in absence of GSH in the medium which precluded the continuation of the culture. At exposure concentrations of 10–35 µg MA the variation of the nominal relative total growth was 67–92% in presence of 10 mM GSH, compared with exceedingly low (culture not continued due to strong cytotoxicity) to 59%, clearly showing protection against MA-provoked cytotoxicity by GSH (Table 1). Table 3A, B show that the cytotoxicity of MA to ML cells is already abrogated by a concentration of 1 mM GSH.

**Table 3:** Cytotoxicity of MA to MLA cells abrogated by 1 mM GSH: A. Absence of GSH and B. Presence of 1 mM GSH

A. Absence of GSH													
Methyl acrylate [µg/ml]			Cell count per mL						Relative growth during treatment (RGDT) (%)	Suspension growth (SG)	Relative suspension growth (RSG) (%)	Relative cloning efficiency (RCE_2_) (%)	Relative total growth (RTG) (%)
			4 h after treatment (× 10^5^)		24 h after treatment (× 10^5^)		48 h after treatment (× 10^5^)						
			Cell count	Mean	Cell count	Mean	Cell count	Mean					
0 (Vehicle control)		A	10.04	10.36	6.35	6.27	11.74	11.32	100.0	17.7	100.0	100.0	100.0
		B	10.67		6.19		10.89						
5.0		A	10.04	9.97	5.48	5.52	10.70	10.71	96.3	14.2	80.2	104.2	83.5
		B	9.90		5.55		10.72						
10.0		A	9.87	9.93	5.13	5.01	11.40	11.17	95.9	13.4	75.6	100.0	75.6
		B	9.99		4.89		10.94						
15.0		A	9.15	9.02	4.46	4.53	10.51	10.47	87.1	10.3	58.2	84.9	49.4
		B	8.89		4.60		10.43						
20.0		A	7.91	8.61	5.03	4.36	9.68	10.01	83.1	9.1	51.2	100.8	51.6
		B	9.31		3.69		10.34						
25.0		A	8.67	8.34	3.65	3.51	8.59	9.16	80.5	6.5	36.4	88.2	32.1
		B	8.01		3.36		9.72						
30.0		A	8.59	8.50	2.78	2.66	6.97	6.58	82.0	3.6	20.2	71.7	14.5
		B	8.40		2.54		6.18						
35.0		A	8.38	8.34	1.86	1.92	n.c.^1^						
		B	8.29		1.98								
MMS 15		A	9.61	9.22	4.89	4.86	8.69	8.15	89.0	8.8	49.7	48.5	24.1
		B	8.82		4.83		7.61						

B. Presence of 1 mM GSH												
Methyl acrylate [µg/ml]		Cell count per ml						Relative growth during treament (RGDT) (%)	Suspension growth (SG)	Relative suspension growth (RSG) (%)	Relative cloning efficiency (RCE_2_) (%)	Relative total growth (RTG) (%)
		4 h after treatment (× 10^5^)		24 h after treatment (× 10^5^)		48 h after treatment (× 10^5^)						
		Cell count	Mean	Cell count	Mean	Cell count	Mean					
0 (Vehicle control)	A	10.94	10.37	7.01	6.96	12.28	12.05	100.0	20.9	100.0	100.0	100.0
	B	9.79		6.90		11.81						
5.0	A	10.03	9.99	6.56	6.59	12.12	11.59	96.4	18.4	87.8	114.4	100.4
	B	9.95		6.61		11.05						
10.0	A	10.04	10.00	6.09	6.24	12.42	12.01	96.5	18.1	86.2	96.3	83.0
	B	9.96		6.38		11.59						
15.0	A	9.45	9.71	6.53	6.52	12.22	11.54	93.6	17.6	84.0	120.3	101.1
	B	9.96		6.50		10.86						
20.0	A	10.23	10.00	6.01	5.95	11.91	11.64	96.4	16.7	79.6	135.2	107.7
	B	9.76		5.88		11.36						
25.0	A	9.92	9.59	6.09	6.16	11.51	11.09	92.5	15.8	75.4	123.5	93.1
	B	9.25		6.23		10.66						
30.0	A	9.14	9.38	6.54	6.26	11.02	11.22	90.4	15.9	75.7	112.6	85.3
	B	9.61		5.97		11.41						
35.0	A	8.73	8.97	6.23	6.19	11.61	11.52	86.5	15.4	73.6	118.3	87.0
	B	9.20		6.14		11.43						

n. c., culture not continued due to strong cytotoxicity; MMS, methyl methanesulfonate

GSH (10 mM), was not present during the exposure to MA, but after this exposure, was not able to abrogate the cytotoxicity of MA. This is best seen at an exposure concentration of 25 µg MA/ml where the nominal relative total growth was only 6%, even less than in the total absence of GSH (27%). After exposure concentrations of 10–35 µg MA the variation of the nominal relative total growth was upon posttreatment with 10 mM GSH 6–73%, clearly much lower compared with the simultaneous presence of GSH during the exposure to MA (67–92%). Thus, reversion of the cytotoxicity of MA is not possible post-treatment with GSH (Table 1).

The use of 2-mercaptoethanol as a potential sulphhydryl-containing substitute for GSH showed that it has in principle also a protective effect against the MA-evoked cytotoxicity, but is less effective. At exposure concentrations of 10–35 µg MA the variation of the nominal relative total growth was in presence of 10 mM 2-mercaptoethanol 28–80% compared with 67–92% in presence of 10 mM GSH (Table 1).

Similar to the above-shown data for MA, Table 2 shows that EA at concentrations between 10 to 35 µg/ml is also cytotoxic to ML cells (dose-dependent decrease of the relative total growth from 69% at 10 µg EA/ml to 1.9% at 35 µg EA/ml). Again, similar to the above-shown data for MA, the simultaneous presence of 10 mM GSH during the exposure period to EA abrogated the cytotoxicity of EA (at concentrations of 10–35 µg EA/ml from a relative total growth of 1.9–69% in absence of GSH to 62–78% upon thw simultaneous presence of GSH). For this protection against the cytotoxicity of EA, GSH had to be present during the phase of exposure to EA since post-treatment with GSH did not prevent cytotoxicity. At concentrations of 10–35 µg EA/ml the relative total growth was between n.c. (culture not continued due to strong cytotoxicity) and 71%, clearly not better than in the total absence of GSH [1.9–69%]). The protective effect of another sulphhydryl-containing compound, 2-mercaptoethanol, was similar to that of GSH (between 10 and 35 µg/ml EA the relative total growth was 42–87% in presence of 2-mercaptoethanol, compared with 62–78% in presence of GSH).

Table 3A, B show that for the protection against the cytotoxicity of MA to ML cells a concentration of 1 mM GSH is sufficient. The same is shown for EA in Table 4A, B.

**Table 4:** Cytotoxicity of EA to MLA cells abrogated by 1 mM GSH: A. Absence of GSH, B. Presence of 1 mM GSH

A. Absence of GSH												
Ethyl acrylate [µg/ml]		Cell count per ml						Relative growth during treatment (RGDT) (%)	Suspension growth (SG)	Relative suspension growth (RSG) (%)	Relative cloning efficiency (RCE 2) (%)	Relative total growth (RTG) (%)
		4 h after Treatment (× 10^6^)		24 h aft er treatment {× 10^6^)		48 h after treatment (× 10^6^)						
		Cell count	Mean	Cell count	Mean	Cell count	Mean					
0 (Vehicle control)	A	9.10	8.95	5.57	5.53	12.63	12.57	100.0	17.4	100.0	100.0	100.0
	B	8.79		5.49		12.50						
5.0	A	8.52	8.58	5.43	5.05	11.61	12.1 5	95.9	14.7	84.7	96.9	82.0
	B	8.64		4.67		12.69						
10.0	A	9.20	8.79	4.97	4.49	11.31	11.42	98.2	12.6	72.5	109.5	79.4
	B	8.37		4.01		11.53						
15.0	A	8.01	8.00	4.75	4.29	10.95	11.37	89.4	10.9	62.8	101.6	63.8
	B	7.99		3.83		11.78						
20.0	A	7.42	7.53	4.17	3.90	11.40	11.58	84.2	9.5	54.6	111.4	60.9
	B	7.64		3.62		11.76						
25.0	A	7.70	7.47	3.65	3.56	10.32	10.26	83.5	7.6	43.9	101.6	44.6
	B	7.24		3.47		10.19						
30.0	A	6.97	7.1 7	3.05	3.14	9.80	9.25	80.2	5.8	33.4	92.4	30 9
	B	7.37		3.22		8.69						
35.0	A	8.39	7.51	2.46	2.39	7.77	8.34	83.9	4.2	24.1	42.2	10.2
	B	6.62		2.32		8.91						
MMS 15	A	8.04	8.30	5.09	4.83	8.73	7.37	92.8	8.2	47.5	52.2	24.8
	B	8.56		4.56		6.01						

B. Presence of 1 mM GSH												
Ethyl acrylate [µg/ml]		Cell count per ml						Relative growth during treament (RGDT) (%)	Suspension growth (SG)	Relative suspension growth (RSG) (%)	Relative cloning efficiency (RCE_2_) (%)	Relative total growth (RTG) (%)
		4 h after treatment (× 10^6^)		24 h after treatment (× 10^6^)		48 h after trea tment (× 10^6^)						
		Cell count	Mean	Cell count	Mean	Cell count	Mean					
Vehicle control	A	8.32	8.19	5.37	5.70	11.93	11.52	100.0	16.4	100.0	100.0	100.0
	B	8.06		6.03		11.11						
5.0	A	8.95	8.35	5.04	5.28	12.41	11.97	102.0	16.1	98.1	103.3	101.3
	B	7.75		5.52		11.52						
10.0	A	7.69	7.85	4.80	5.02	12.12	11.62	95.8	14.0	85.1	102.4	87.1
	B	8.01		5.23		11.12						
15.0	A	7.93	8.12	5.18	5.31	11.15	11. 31	99.1	14.9	90.5	88.4	80.0
	B	8.30		5.43		11.46						
20.0	A	7.31	7.76	5.43	5.28	12. 19	12.12	94.7	15.1	92.2	106.7	98.4
	B	8.21		5.12		12.04						
25.0	A	7.11	7.65	503	4.89	12.29	12.56	93.4	14.3	87.3	100.0	87.3
	B	8.19		4.75		12.82						
30.0	A	6.95	7.61	6.06	5.64	11.43	11.46	92.9	15.0	91. 3	101.6	92.8
	B	8.27		5.21		11.48						
35.0	A	6.18	6.8 4	6.76	6.22	11.43	11.44	83.5	14.8	90.4	95.4	86.2
	B	7.49		5.68		11.44						

MMS, methyl methanesulfonate

The data prove that the sulphhydryl-containing tripeptide GSH provides an efficient protection against the cytotoxicity of MA and EA to ML cells. The cytotoxicity of MA and EA may be caused by the depletion of GSH or by the direct interaction of these α,β-unsaturated electrophilic carbonyls with nucleophilic cellular constituents other than GSH or (most likely) both. The lack of reversion of MA’s and EA’s cytotoxicity by post-treatment with GSH implies that the exposure of the cells to MA and EA has caused irreversible damage.

### Mutagenicity of MA and EA abrogated by GSH

**Fig. 1:**
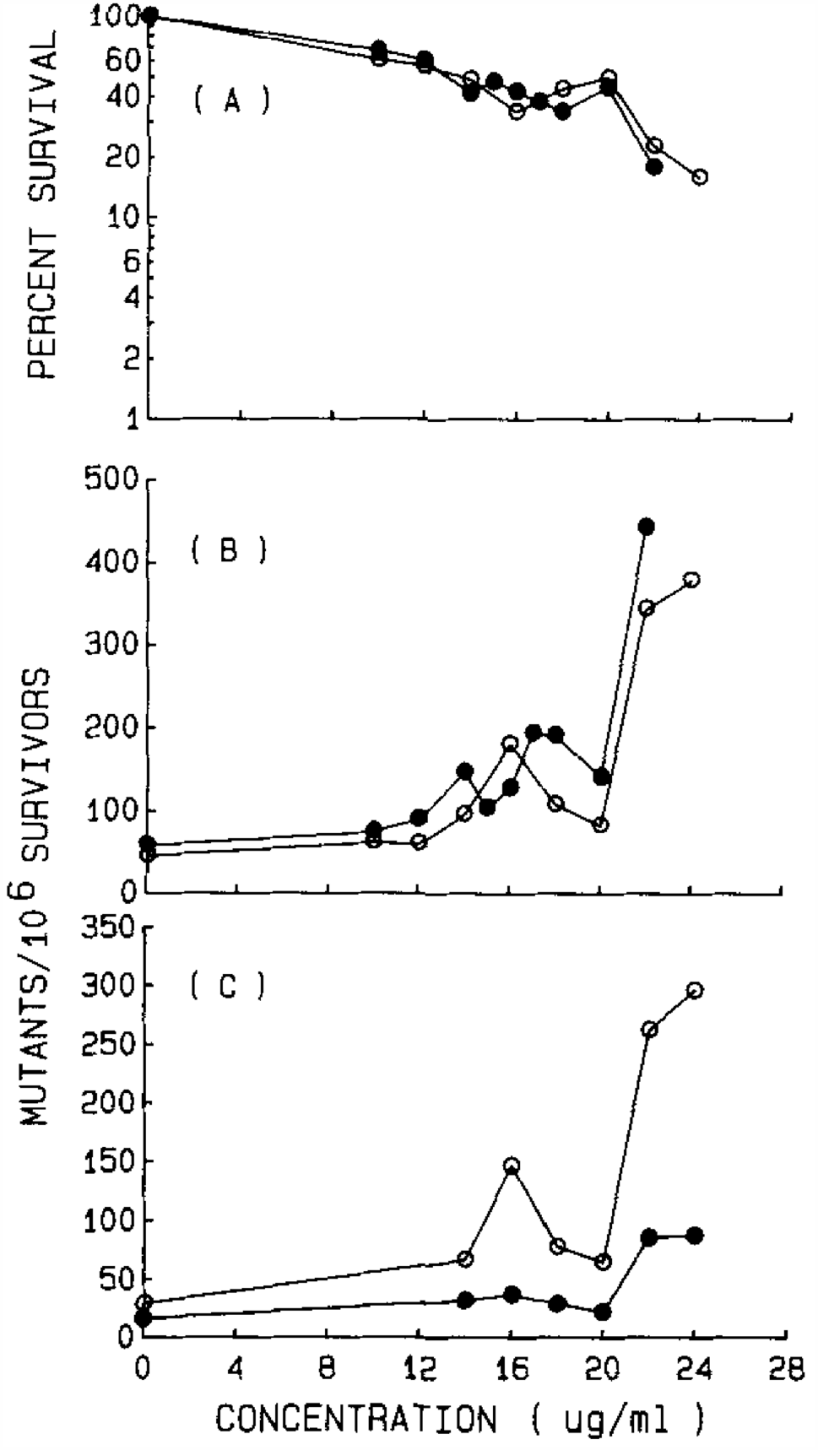
Cytotoxicity (**A**) and mutagenicity (**B**) of methyl acrylate to L5178Y/TK^+/−^ mouse Iymphoma cells when tested without exogenous activation. Results are from two separate experiments and are shown as open or closed symbols, respectively. **C** The small- and large-colony mutant frequencies are shown separately. Open symbols represent the small-colony mutant frequency. Closed symbols represent the large-colony mutant frequency. From Moore et al. 1988. Reprinted with permission

**Fig. 2:**
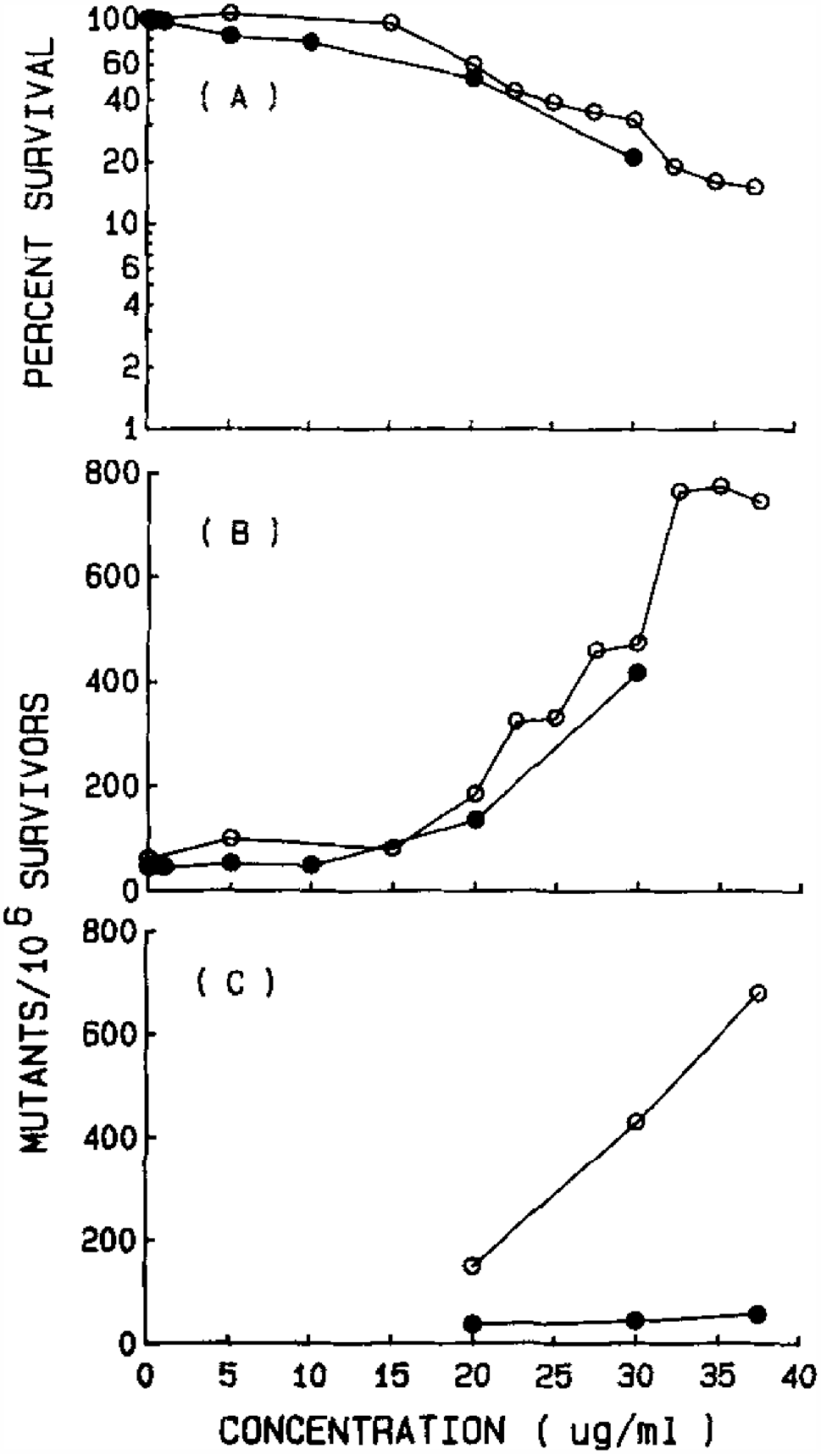
Cytotoxicity (**A**) and mutagenicity (**B**) of ethyl acrylate to L5178Y/TK^+/−^ mouse lymphoma cells when tested without exogenous activation. Results are from two separate experiments and are shown as open or closed symbols, respectively. **C** The small- and large-colony mutant frequencies are shown separately. Open symbols represent the small-colony mutant frequency. Closed symbols represent the large-colony mutant frequency. From Moore et al. 1988. Reprinted with permission

Moore et al. (1988) had shown that MA (Fig. 1) and EA (Fig. 2) are mutagenic to L5178Y/TK^+/−^ mouse lymphoma cells (at 14 and 20 µg/ml, respectively). As can be seen from the present data in presence of 1 mM GSH neither MA (5–35 µg/ml) (Table 5) nor the same concentrations of EA (Table 6) lead to mutagenicity for L5178Y/TK^+/−^ mouse lymphoma cells.

**Table 5:** No mutagenicity of MA in presence of 1 mM GSH

Methyl acrylate [µg/ml]		Number of empty wells^a^			Mutant frequency [per 10^6^ cells]		Colony counts (%) (Absolute counts)					
		Plate 1	Plate 2	Mean	Corrected MF^b^	Corrected mean MF^b^	Small colonies			Large colonies		
Vehicle control	A	80	85	82.5	**101.3**	**70.4**	14	8	**83**	3	3	**17**
	B	83	90	86.5	**46.5**		13	6	**(10)**	2	0	**(2)**
5	A	71	77	74.0	**128.5**	**98.5**	17	10	**65**	9	11	**35**
	B	86	79	82.5	**71.2**		9	15	**(13)**	3	3	**(7)**
10	A	73	84	78.5	**119.7**	**93.8**	14	14	**69**	11	1	**31**
	B	85	84	84.5	**70.4**		10	6	**(11)**	2	6	**(5)**
15	A	88	72	80.0	**77.2**	**66.7**	5	12	**64**	3	13	**36**
	B	89	83	86.0	**54.3**		6	14	**(9)**	1	1	**(5)**
20	A	77	75	76.0	**95.3**	**73.1**	14	11	**65**	7	11	**35**
	B	84	85	84.5	**52.0**		9	11	**(11)**	4	1	**(6)**
25	A	84	84	84.0	**64.9**	**57.0**	9	10	**82**	3	2	**18**
	B	84	86	85.0	**49.6**		9	9	**(9)**	3	1	**(2)**
30	A	85	82	83.5	**68.9**	**75.7**	11	14	**87**	2	1	**13**
	B	84	78	81.0	**82.5**		11	16	**(13)**	2	4	**(2)**
35	A	86	88	87.0	**46.3**	**49.9**	8	7	**80**	2	1	**20**
	B	84	87	85.5	**53.5**		9	9	**(8)**	3	0	**(2)**

^**a**^Number of wells without colonies 10 days after seeding about 2000 cells/well into selection medium

^b^Mutation frequency corrected on the basis of the absolute cloning efficiency 2 (CE_2_) at the end of the expression period

**Table 6:** No mutagenicity of EA in presence of 1 mM GSH

Ethyl acrylate [µg/ml]		Number of empty wells^a^			Mutant frequency [per 10^6^ cells]		Colony counts (%) (absolute counts)					
		Plate 1	Plate 2	Mean	Corrected MF^b^	Corrected Mean MF^b^	Small colonies			Large colonies		
Vehicle control	A	89	86	87.5	**48.0**	**62.1**	3	9	**75**	4	2	**25**
	B	80	85	82.5	**76.1**		14	8	**(9)**	3	3	**(3)**
5	A	82	81	81.5	**70.6**	**77.8**	12	14	**80**	3	3	**20**
	B	84	81	82.5	**84.9**		10	11	**(12)**	2	4	**(3)**
10	A	75	79	77.0	**107.2**	**92.3**	23	16	**84**	2	4	**16**
	B	85	80	82.5	**77.3**		10	15	**(16)**	4	2	**(3)**
15	A	87	83	85.0	**77.9**	**61.8**	13	16	**83**	1	0	**17**
	B	84	91	87.5	**48.0**		8	2	**(10)**	4	3	**(2)**
20	A	85	81	83.0	**81.5**	**51.2**	14	14	**82**	1	2	**18**
	B	89	90	89.5	**28.1**		3	4	**(9)**	4	2	**(2)**
25	A	77	81	79.0	**93.1**	**80.4**	18	16	**81**	4	0	**19**
	B	80	90	85.0	**66.1**		13	5	**(13)**	5	1	**(3)**
30	A	85	81	83.0	**76.6**	**80.6**	10	17	**72**	1	3	**28**
	B	79	82	80.5	**84.2**		15	8	**(13)**	7	8	**(5)**
35	A	85	82	83.5	**73.4**	**100.8**	10	19	**75**	4	0	**25**
	B	70	81	75.5	**130.4**		19	10	**(15)**	9	6	**(5)**

^**a**^Number of wells without colonies 10 days after seeding about 2000 cells/well into selection medium

^b^Mutation frequency corrected on the basis of the absolute cloning efficiency 2 (CE_2_) at the end of the expression period

Upon treatment with 5–35 µg MA/ml the mean corrected MFs were between 49.9 and 98.5 per 10^6^ cells compared with 70.4 in the vehicle control. At the highest concentration of MA (35 µg/ml) the mean corrected MF was 49.9 per 10^6^ cells. The mean corrected MFs at all MA concentrations (49.9–98.5 per 10^6^ cells) were below the respective calculated threshold for a mutagenic effect based on the GEF (126 plus the control value = 196 mutant colonies per 10^6^ cells). No dose-related increase in the corrected MFs was observed as determined by testing for linear trend: The slope was −1.79647 and the one-sided *p*-value was 0.953 (statistical significance at a significance level of 5% is reached if the slope is greater than 0 and the one-sided *p*-value is lower than 0.05).

The same pertained to the treatment of mouse lymphoma TK^+/−^ cells with EA: Upon treatment with 5–35 µg EA/ml the mean corrected MFs were between 51.2 and 100.8 per 10^6^ cells compared with 62.1 in the vehicle control. The mean corrected MFs at all EA concentrations were below the respective calculated threshold for a mutagenic effect based on the GEF (188 mutant colonies per 10^6^ cells). No dose-related increase in the corrected MFs was observed as determined by testing for linear trend: The one-sided *p*-value was 0.1351 (statistical significance at a significance level of 5% is reached if the one-sided *p*-value is lower than 0.05).

The separate counting of small colonies (slowly growing, indicative of chromosome mutations) and large colonies (fast growing, indicative of gene mutations) also showed no significant increase in the number of colonies (absolute numbers) upon treatment with MA or EA (64–87 small colonies upon treatment with 5–35 µg MA, 72–84 upon treatment with the same doses of EA, neither one of them dose-related, compared with 83 and 75 in the vehicle controls, respectively; 13–36 large colonies upon treatment with 5–35 µg MA, 16–28 upon treatment with the same doses of EA, neither one of them dose-related, compared with 17 and 25 in the vehicle controls, respectively (Tables 5, 6)).

The positive control substance MMS (15 µg/ml) led, as expected, to clearly increased mutant frequencies (mean MFcorr.: 916.9 and 1142 in the experiment with MA and EA, respectively). The values largely exceeded the respective calculated thresholds for the mutagenic effects based on the GEF (126 plus the MF of the respective negative controls, 70.4 and 62.1 per 10^6^ cells, respectively), clearly proving the required sensitivity of the test. In addition, the corrected MFs were comparable with the historical positive control (MMS 15 µg/ml) data range (January 2011–March 2019: 256.5–1496.6, mean 803.6, standard deviation 274.6, number of experiments 36 performed in our laboratory at BASF). Besides, the obtained values fulfilled the criteria for positive controls as stated in the current OECD Guideline 490.

The vehicle control values (corrected mean MF 70.4 and 62.1 per 10^6^ cells in the experiments with MA and EA, respectively) also were within the control values for the same concentration (1%, v/v) of the same vehicle (DMSO) (including the data for acetone as a vehicle) in the mouse lymphoma TK^+/−^ tests without S9 mix: 23.3–109.2 per 10^6^ cells, mean 50.9, standard deviation 18.1, 33 experiments performed between January 2011 and March 2019 in our laboratory at BASF.

Osmolality and pH values were not relevantly influenced by test substance treatment. In the absence and presence of 1 mM GSH no precipitation in the culture medium was observed up to the highest applied test substance concentration.

Thus, the data clearly showed that 1 mM GSH abrogated the mutagenicity of MA and EA which had been reported by Moore et al. (1988) in the same mutagenicity test (mouse lymphoma TK^+/−^) already at lower MA and EA doses (14 and 20 µg/ml, respectively) than those used in the herewith presented studies (5–35 µg/ml).

### Recovery of residual acrylates and GSH at the end of the exposure period in the cytotoxicity and mutagenicity experiments

Tables 7 and 8 show that the concentration of GSH which was sufficient to abrogate the cytotoxicity and mutagenicity of MA and EA, 1 mM, had dramatically reduced the residual concentration of MA (from a recovery of 38–41% to 1.2–1.3% of the initially added concentration) and of EA (from 51–55 to 1.3–2.1%) at acrylate concentrations of 20, 25 and 35 µg/ml. This shows that 1 mM GSH reduced the concentrations of MA and EA to minimal levels implying that this extensive reduction of MA and EA in all likelihood was the mechanism by which the cytotoxicity and mutagenicity of MA and EA in the mouse lymphoma cells was abrogated.

**Table 7:** Methyl acrylate and glutathione recoveries in the end of the mutagenicity experiments

Methyl acrylate initially added [µg/ml]	Glutathione initially added [µM]	Methyl acrylate finally recovered^a^ [µg/ml]/%^c^/%^d^	Glutathione finally recovered^b^ [µmol/g cell lysate]
0	0	˂0.2	59.6
20	0	7.6/38	21.9
25	0	9.7/39	12.2
35	0	14.4 /41	10.9
0	10	˂0.2	37.3
20	10	6.7/33^c^/88^d^	15.4
25	10	8.3/33/86	11.7
35	10	14.1/40/98	8.12
0	100	˂0.2	48.3
20	100	4.6/23/61	24.4
25	100	6.0/24/62	13.9
35	100	11.1/32/77	10.5
0	1000	˂0.2	49.6
20	1000	0.24/1.2/3.2	55.6
25	1000	0.31/1.2/3.2	52.8
35	1000	0.47/1.3/3.3	60.4

^a^In the end of the mutagenicity experiment recovered in the culture supernatant (in the culture cell lysate ˂1 µg/ml)

^b^In the end of the mutagenicity experiment recovered in the culture cell lysate (in the culture supernatant below detection)

^c^Compared with the initially added amount

^d^Compared with the residual amount after the exposure period in absence of GSH

**Table 8:** Ethyl acrylate and glutathione recoveries in the end of the mutagenicity experiments

Ethyl acrylate initially added [µg/ml]	Glutathione initially added [µM]	Ethyl acrylate finally recovered^a^ [µg/ml]/%^c^/%^d^	Glutathione finally recovered^b^ [µmol/g cell lysate]
0	0	˂0.2	53.3
20	0	11/55	9.28
25	0	13/52	8.39
35	0	18/51	5.55
0	10	˂0.2	56.25
20	10	7.5/37^c^/68^d^	16.51
25	10	m^e^	10.98
35	10	13/37/72	6.22
0	100	˂0.2	65.02
20	100	5.3/26/48	22.83
25	100	6.4/26/49	17.30
35	100	8.9/25/49	14.48
0	1000	˂0.2	48.08
20	1000	0.27/1.3/2.5	84.74
25	1000	0.48/1.9/3.7	82.12
35	1000	0.73/2.1/4.1	82.70

^a^In the end of the mutagenicity experiment recovered in the culture supernatant (in the culture cell lysate ˂1 µg/ml)

^b^In the end of the mutagenicity experiment recovered in the culture cell lysate (in the culture supernatant 0, except after the initial addition of 1000 µM GSH 4.15, 2.05, 2.97 and 3.05 µmol/g cell lysate after co-incubation with 0, 20, 25 and 35 µg ethyl acrylate/ml, respectively

^c^Compared with the initially added amount

^d^Compared with the residual amount after the exposure period in absence of GSH

^e^Mistake

Moreover, Tables 7 and 8 show that the reduction of the cytotoxicity and mutagenicity of MA and EA to the mouse lymphoma cells was dose-dependently related to the concentrations of GSH. 10 µM GSH reduced MA recovered after the exposure period only minimally (from 38–41% to 33–40%), that of EA only slightly (from 51–55% to 37%), 100 µM GSH substantially more (from 38–41% to 23–32% and from 51–55% to 25–26%, respectively), and 1000 µM GSH dramatically (from 38–41% to 1.2–1.3% and from 51–55% to 1.3–2.1%). It may therefore be expected that the even higher in vivo levels of GSH in the mouse liver (7.55 ± 0.57 µmol/g tissue in the homogenate, see next chapter) and mouse stomach (2.84 ± 0.22 µmol/g tissue in the homogenate) likewise substantially reduce MA and EA levels (for verification see next chapter).

### Glutathione (GSH) in mouse liver and forestomach not depleted by methyl acrylate (MA) or ethyl acrylate (EA)

Four male C57BL/6 mice were orally treated with 0, 20, 50 and 100 mg/kg MA or EA. Three hours later the animals were killed and the GSH levels were determined in their liver and forestomach.

Table 9 shows that the GSH levels in the liver were 9.22 ± 2.23, 7.66 ± 0.62 and 7.17 ± 0.84 µmol/g tissue after treatment with 20, 50 and 100 mg/kg MA, respectively, and 8.34 ± 0.46, 7.67 ± 0.58 and 8.03 ± 0.71 after treatment with 20, 50 and 100 mg/kg EA, respectively, compared with 7.55 ± 0.57 µmol/g in the vehicle (drinking water) control. GSH levels in the forestomach were 3.60 ± 0.33, 3.59 ± 0.63 and 2.81 ± 0.31 µmol/g after treatment with 20, 50 and 100 mg/kg MA, respectively, and 2.66 ± 0.73, 2.60 ± 0.54 and 3.11 ± 0.33 µmol/g after treatment with 20, 50 and 100 mg/kg EA compared with 2.84 ± 0.22 µmol/g in the vehicle control.

**Table 9:** Glutathione levels in the liver and forestomach of mice orally treated with methyl acrylate or ethyl acrylate

Treatment	Dose [mg/kg bw]	Tissue [µmol/g tissue]	Glutathione^a^
Vehicle (water)		Liver	7.55 ± 0.57
Methylacrylate	20	9.22 ± 2.23	
	50	7.66 ± 0.62	
	100	7.17 ± 0.84	
Ethylacrylate	20	8.34 ± 0.46	
	50	7.67 ± 0.58	
	100	8.03 ± 0.71	
Vehicle (water)		Forestomach	2.84 ± 0.22
Methylacrylate	20	3.60 ± 0.33	
	50	3.59 ± 0.63	
	100	2.81 ± 0.31	
Ethylacrylate	20	2.66 ± 0.73	
	50	2.60 ± 0.54	
	100	3.11 ± 0.33	

^a^Mean ± standard deviation

Hence, there is clearly no depletion, nor reduction of GSH by treatment with up to 100 mg/kg MA or EA under in vivo conditions.

## Conclusion

Moore et al. (1988) had shown that MA and EA are mutagenic to mouse lymphoma L5178Y/TK^+/−^ cells. Mutagenicity was observed starting at concentrations of 14 and 20 µg/ml, respectively.

The present investigations confirmed the mutagenicity and cytotoxicity of MA and EA to the mouse lymphoma L5178Y/TK^+/−^ cells observed by Moore et al. (1988). However, the present investigations showed that the limited availability of GSH under these test conditions seem to be responsible for this outcome and the addition of GSH was able to abrogate the cytotoxicity (Tables 1, 2, 3, 4) and the mutagenicity (Tables 5, 6) to these mouse lymphoma cells. 1 mM GSH was sufficient to abrogate both, the cytotoxicity and the mutagenicity indicating that MA and EA are not mutagenic to the mouse lymphoma L5178Y/TK^+/−^ cells in presence of 1–10 mM GSH. Analysis of the residual MA and EA after the exposure period of the mutagenicity and cytotoxicity assays (co-incubation of the acrylates with GSH) showed reductions of the residual acrylates which were related to the GSH concentrations in a dose-dependent manner and reached at 1 mM GSH a reduction of MA and EA of 97% and 96–97%, respectively. Therefore, it is expected that even higher in vivo GSH levels in the mouse liver (7.55 ± 0.57 µmol/g tissue in homogenate) and forestomach (2.84 ± 0.22 µmol/g tissue in homogenate) may efficiently protect against the cytotoxicity and mutagenicity of MA and EA up to the highest tested dose of 100 mg/kg body weight which did not lead to any reduction of the in vivo GSH levels in these organs (Table 9). A possible, but unknown enzymatic contribution of mouse glutathione S-transferases to the detoxication of MA and/or EA would even add to the in vivo protection by GSH. It is, furthermore, unknown whether GSH conjugation with MA and/or EA may occur more rapidly than ester cleavage and how the GSH conjugation rates of the esters would compare with those of the liberated acrylate. In any event, the data obtained in this study lead to the conclusion that the in-situ levels of GSH in mouse liver and forestomach can efficiently protect against MA and EA-induced mutagenicity in vivo.
